# Supplementary material for: Venomix: a simple bioinformatic pipeline for identifying and characterizing toxin gene candidates from transcriptomic data
Source: PeerJ. 2018 Jul 31;6:e5361. doi: 10.7717/peerj.5361 (PMC6074769; doi:10.7717/peerj.5361)
Supplement: Supplemental Information 3 [file peerj-06-5361-s003.gz › FinalOutput_E-6/Turripeptide_Ici9/finaltree.pdf]

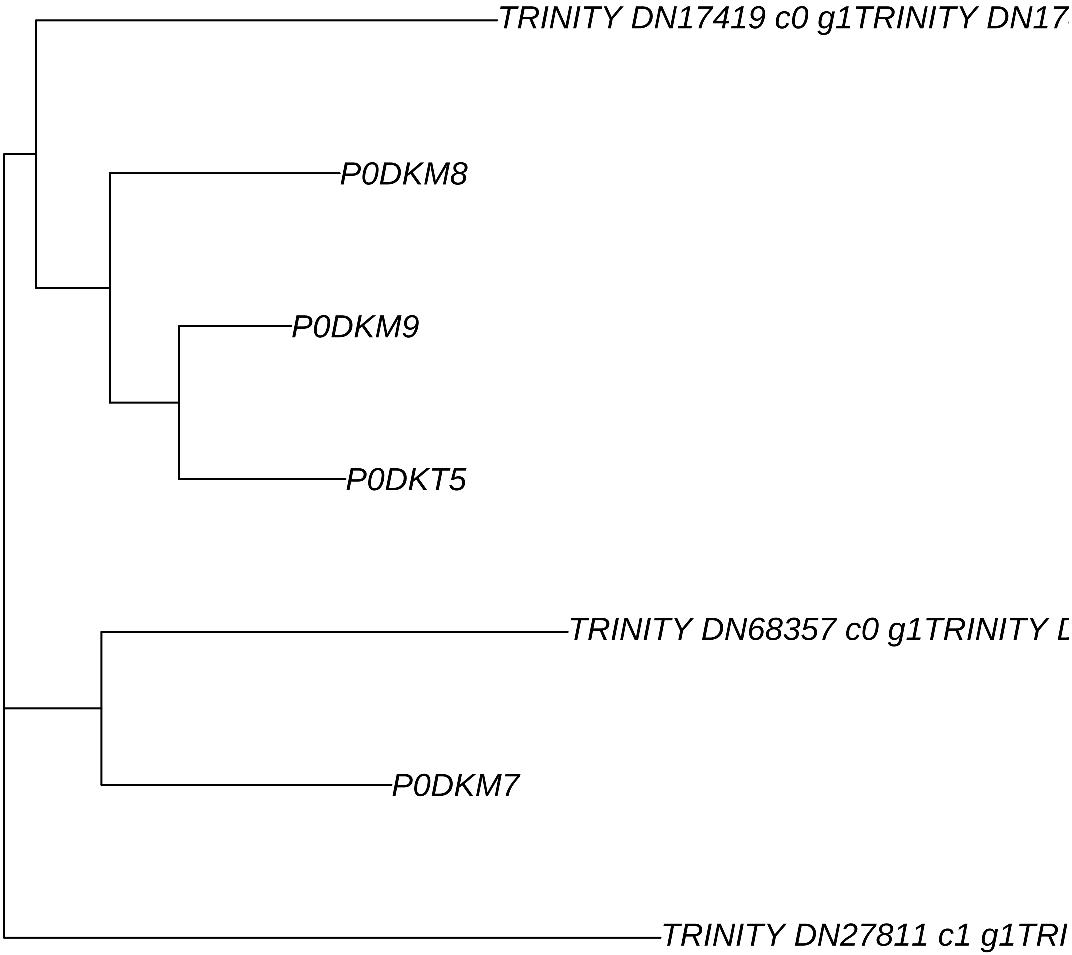
TRINITY DN17419 c0 g1TRINITY DN17
P0DKM8
P0DKM9
P0DKT5
TRINITY DN68357 c0 g1TRINITY D
P0DKM7
TRINITY DN27811 c1 g1TRI
